# A Promising Candidate: Heparin-Binding Protein Steps onto the Stage of Sepsis Prediction

**DOI:** 10.1155/2019/7515346

**Published:** 2019-12-16

**Authors:** Yang Yang, Guihuan Liu, Qingnan He, Jie Shen, Linyong Xu, Ping Zhu, Mingyi Zhao

**Affiliations:** ^1^Department of Pediatrics, The Third Xiangya Hospital, Central South University, Changsha, Hunan Province 410013, China; ^2^Xiangya School of Medicine, Central South University, Changsha, Hunan Province 410013, China; ^3^Guangdong Cardiovascular Institute, Guangdong Provincial People's Hospital, Guangdong Academy of Medical Sciences, Guangzhou, Guangdong 510100, China; ^4^Department of Biomedical Informatics, School of Life Sciences, Central South University, Changsha, Hunan 410000, China

## Abstract

Sepsis is a systemic inflammatory response syndrome caused by infection. With high morbidity and mortality of this disease, there is a need to find early effective diagnosis and assessment methods to improve the prognosis of patients. Heparin-binding protein (HBP) is a granular protein derived from polynuclear neutrophils. The biosynthetic HBP in neutrophils is rapidly released under the stimulation of bacteria, resulting in increased vascular permeability and edema. It is reasonable to speculate that the HBP in plasma may serve as a novel diagnostic marker for sepsis, bacterial skin infection, acute bacterial meningitis, leptospirosis, protozoan parasites, and even some noncommunicable diseases. It implies that in the detection and diagnosis of sepsis, it will be possible to make relevant diagnosis through this new indicator in the future. In this review, we summarize the typical biological function of HBP and its latest research progress to provide theoretical basis for clinical prediction and diagnosis of sepsis.

## 1. Introduction

Infectious diseases continue to pose a significant problem for healthcare, as a number of these diseases have high morbidity and mortality due to their vague early symptoms. Sepsis is a condition that arises as a result of severe infection, and it is a common cause of mortality in hospitals. Approximately 20%-30% of patients suffering from severe sepsis, however, do not exhibit typical signs such as organ dysfunction upon hospital admission; instead, the condition deteriorates to severe sepsis within the first 24 hours postadmission [1, 2]. Given this, there is a need to identify an early diagnostic biomarker for sepsis.

The typical biomarkers used to identify infectious diseases, including IL-6, C-reactive protein (CRP), white blood cell count (WBC), and lactate, display a similar pattern where they exhibit higher levels in patients with severe sepsis; however, there are substantial overlaps among various patient groups [3]. Even procalcitonin, the most frequently evaluated biomarker among more than 100 markers proposed for the use as infection markers, still exhibits substantial differences when used to determine mortality rates, and these differences can range from 7.5% to 10% [4]. Based on this, none of these biomarkers are adequate for routine clinical use in diagnosing sepsis.

Heparin-binding protein (HBP), also known as azurocidin or cationic antimicrobial protein of 37 kDa (CAP 37), is a member of the serine proteinase derived from the polymorphonuclear neutrophil (PMN) family [5]. Initially, this protein gained attention due to its antimicrobial properties [6], and later, it was determined that HBP acted as a multifunctional mediator in infection and inflammation. HBP is prefabricated in PMNs [7] and released rapidly after stimuli by various bacterial structures [8–11], cytokines, inflammation factors, and chemotactic factors [12]. When released, HBP can exert significant subsequent effects on the immune system. It is a potent chemoattractant for many types of cells, particularly monocytes [13], and it is a powerful inducer of vascular leakage and edema formation [14]. HBP is also secreted following the extravasation of PMNs, where it interacts with other cell types such as corneal epithelial cells [15] and smooth muscle cells [16] to facilitate similar biological functions. These characteristics make HBP a promising candidate for use in the detection of early infection. Here, we review the latest research progress in regard to HBP to provide new ideas for improving the clinical prediction and diagnosis of sepsis.

## 2. HBP Molecular Structure and Mechanisms

### 2.1. The Molecular Structure of HBP

Heparin-binding protein (HBP) is a member of the serine proteinase family that possesses 221 or 222 amino acid residues [5]. This protein exhibits a 47% direct sequence similarity to human elastase [5], and both proteins possess eight strictly conserved cysteine residues that form disulfide bridges [13]. HBP, however, is typically thought to be devoid of serine proteinase activity [17] due to the mutations of 2 residues within the catalytic triad. Most serine proteinases contain a catalytic center composed of His, Asp, and Ser [18]; however, in HBP, His 41 and Ser 175 are substituted by Ser and Gly, respectively [13]. Similar substitutions occur in haptoglobin [19], protein Z [20], and hepatocyte growth factor [21]. Despite a lack of serine proteinase activity, reports suggest that HBP may possess the ability to cleave certain insulin-like growth factor-binding proteins (IGFBP-1, IGFBP-2, and IGFBP-4) to modulate inflammation and wound healing [22]. One of the mutations, where the histidine at position 41 is mutated to serine and thus exposed at the surface of the molecule, has been demonstrated to significantly influence the antimicrobial role of HBP [23] and the ability of this protein to bind to monocytes [24]. Specifically, the release of IL-6 from monocytes induced by LPS is enhanced up to 10-fold by the aa 20-44 HBP peptide, and these effects are abolished in the presence of this position 41 mutation [24]. Another structure of HBP that is associated with its inducing effects is N-linked glycosylation. HBP possesses three potential N-linked glycosylation sites (-Asn-X-Thr-) at residues 100, 114, and 145 [13]. The putative fourth carbohydrate attachment site is located in the region between residues 89 and 114 [5]. The biological activity of HBP in regard to mediating LPS-induced IL-6 release from monocytes is severely reduced by the removal of N-glycosylation [25]. Interestingly, however, the loss of HBP glycosylation does not affect the folding, secondary and tertiary structure, or stability of HBP [25]. It should also be noted that HBP is a fundamental molecule that possesses a 12% content of basic residues [5]. The three-dimensional structure of HBP reveals a highly cationic region composed of sixteen basic residues that are concentrated at one pole of the molecule [26]. This cationic region has been demonstrated to be associated with ligand binding and antimicrobial activity [27]. Heparin, a ligand of HBP, binds to the HBP N-terminal region that consists of at least two clusters of basic residues, and once HBP is bound to heparin, its antimicrobial activity is inhibited [27]. The other domains of HBP that are capable of binding other cellular receptors, however, are still exposed after heparin binding [5].

### 2.2. The Immunological Function and Biomarker Plausibility of HBP

HBP was originally discovered in 1984 as a result of its potent antibacterial activity against Gram-negative pathogens [6, 28]. Later, it was reported that a number of Gram-positive organisms, such as *Streptococcus pyogenes* and *Listeria monocytogenes*, were also sensitive to HBP [23]. A number of studies have demonstrated that the peptide 20-44 portion of HBP plays an essential role in its antimicrobial activity by binding to LPS directly [23, 29, 30]. Additionally, as a multifunctional innate-immune defense molecule, HBP exerts wider-ranging influences in the context of host defense. HBP is prefabricated in PMNs and stored in secretory vesicles closed to the membrane [7]. An 89% portion of the total HBP can initially be rapidly released into the environment by exocytosis when PMNs are stimulated by certain bacterial structures, and this release is much greater than that of any other neutrophil granule proteins [31]. The concentration of HBP is clearly elevated in response to a number of infectious diseases such as influenza A (H1N1) infection [14], leptospirosis [32], sepsis [33], and acute kidney injury caused by sepsis [34]. Additionally, the level of HBP generally correlates with the degree of disease severity. Given this, HBP may act as an excellent candidate to predict early infectious diseases. HBP also exhibits high affinity for the N-terminal domain of PTX3, a type of pattern recognition receptor (PRR). The binding of HBP to PTX3 triggers innate immunity responses through a calcium-dependent pathway [35]. Increased endothelial permeability is a common phenomenon in infection, and the specific mechanisms of HBP-induced vascular leakage are described in detail below. Interestingly, neutrophils have been reported to both reduce and increase endothelial permeability *in vitro* [36]. Further research is required to determine if HBP exerts a similar dual effect on vascular leakage.

### 2.3. The Release Mechanisms of HBP

**Figure 1:**
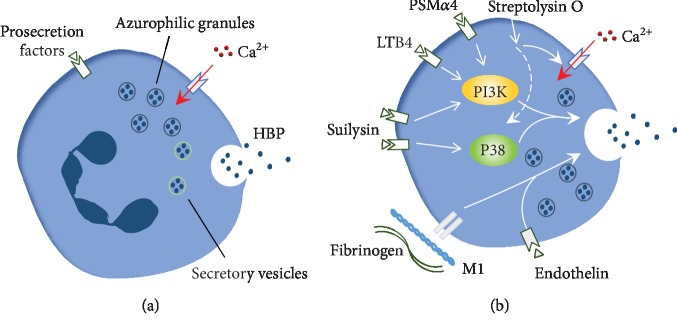
The complex mechanisms underlying HBP release. (a) Prosecretion factors stimulate PMNs to release HBP following an influx of Ca^2+^. (b) The specific signaling pathways of different factors responsible for activating PMNs, including PSM*α*4/LTB4/suilysin-receptor-PI3K, suilysin-GPCR-P38, fibrinogen-M1-*β* integrin, streptolysin O-P38/Ca^2+^ influx, and endothelin-ET receptor.

HBP is an essential mediator of the inflammation reaction, and many factors, such as antigens, cytokines, enzymes, and inflammation factors, can stimulate PMNs to secret HBP [12]. Typically, the release of HBP is caused by a calcium influx-dependent degranulation; however, different factors also modulate unique pathways to promote HBP secretion (Figure 1). Phenol-soluble modulin *α*4 (PSM*α*4) derived from *Staphylococcus aureus* binds to formyl peptide receptor 2 (FPR2) on the surface of PMNs to active PI3K signaling pathways to induce HBP release [8]. LTB4 and suilysin derived from *Streptococcus suis* both similarly trigger PI3K pathways through their interactions with the BLT1 and TLR4 receptors, respectively [9, 37]. Additionally, G-protein-coupled receptors and p38 MAPK are also involved in the release of HBP induced by suilysin [9]. The M1 protein derived from *Streptococcus pyogenes* forms complexes with fibrinogen, and by binding to *β* integrins at the surface of PMNs, these complexes stimulate the cells to secrete HBP [11]. Streptolysin O derived from *Streptococcus pyogenes* perforates PMNs directly, resulting in an influx of Ca(2+) and p38 MAPK activation [38]. It has been confirmed that the infusion of endothelin-1 (ET-1) increases the plasma level of HBP, and dual ET-receptor antagonists markedly counteract this effect during porcine endotoxemia [39]. In human PMNs, the presence of ET receptors has been demonstrated [40], and a similar interaction may exist in human infectious diseases. Further studies indicate that there is a unique variation in neutrophil responses against various stimuli. For example, streptococcal strains induced a markedly higher release of HBP compared to that induced by *Staphylococcus aureus* or *E. coli* [10]. PMNs are generally considered to be the only cells that release HBP, but Schou et al. found that monocytes were also capable of releasing HBP in small amounts, and these amounts were increased in the presence of LPS [24]. Taken together, these observations suggest that the mechanisms underlying HBP release are complicated and that further studies are required to more clearly define these underlying mechanisms.

### 2.4. HBP Induces Vascular Leakage

**Figure 2:**
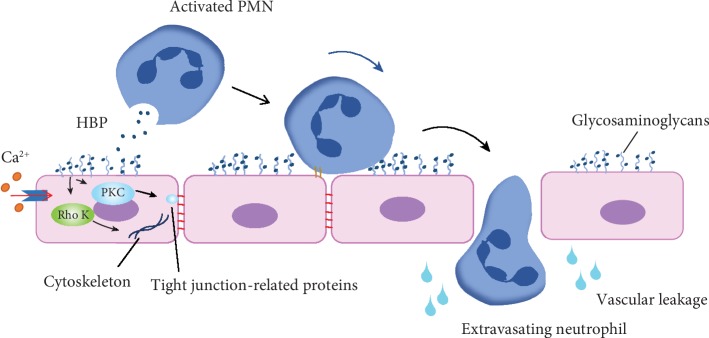
HBP induces increased endothelial permeability that eventually results in vascular leakage. PMNs bind ECs to release HBP, and then HBP interacts with ECs through glycosaminoglycans. Once HBP is internalized via a receptor-mediated process, PKC and Rho kinase are activated, ultimately leading the rearrangement of the cytoskeleton and cell contraction. PKC also phosphorylates tight junction-related proteins to facilitate changes in the tight junctions between ECs. As a result, gaps form between ECs that ultimately result in vascular leakage.

Vascular leakage is a common response to various infectious diseases, and this leakage is followed by infiltration of neutrophils and monocytes. Past studies have indicated that HBP plays an essential role in increasing vascular endothelial permeability [14] (Figure 2). Experimental evidence has demonstrated that intravitreal injection with HBP (20 *μ*g) induced a 6.8-fold increase in vascular permeability [41], and the removal of HBP rendered the postsecretory supernatant of PMNs completely inactive in regard to the ability to induce EC permeability changes [30]. When PMNs adhere to the endothelial lining, *β*2 integrin signaling triggers the release of HBP. HBP interacts directly with the endothelium by binding to glycosaminoglycans present on proteoglycans, and it is then internalized via a receptor-mediated process that requires an intact and functional cytoskeleton [42]. HBP then activates protein kinase C (PKC) and Rho kinase to induce calcium influx into the cells [43, 44], ultimately leading to cytoskeletal rearrangement and cell contraction [30]. Certain studies have demonstrated that PKC exerts distinct effects on dynamic changes within tight junctions, and its activation results in phosphorylation and redistribution of tight junction-related proteins [45]. The Rho-mediated F-actin ring aids in maintaining endothelial barrier integrity during leukocyte diapedesis [46], but its activation by permeability factors is also necessary for vascular leakage [47]. These events result in the formation of gaps between the endothelia, ultimately resulting in vascular leakage and neutrophil extravasation [30].

### 2.5. HBP Chemotactic Role

**Figure 3:**
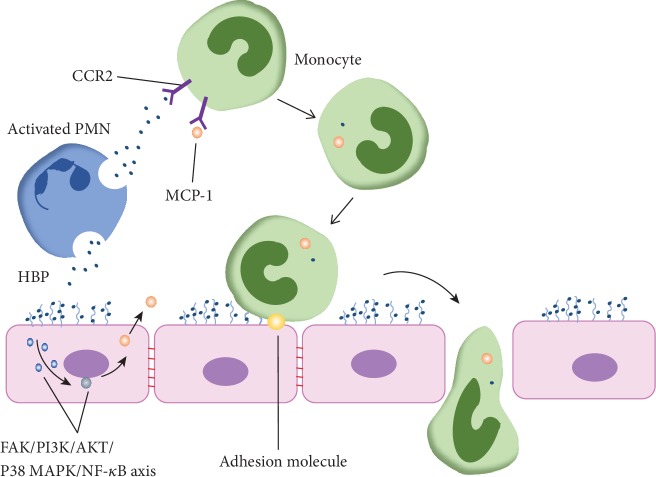
HBP is a potent chemoattractant factor for monocytes. This protein can interact with CCR2 to active monocytes directly, or it can trigger the FAK-PI3K-AKT-P38 MAPK-NF-*κ*B axis in ECs to facilitate the secretion of MCP-1, the ligand of CCR2, to induce monocytes to migrate. Additionally, HBP upregulates ICAM, VCAM, and E-selectin in the ECs, ultimately contributing to the adhesion between ECs and monocytes.

Inflammation is characterized by an early influx of PMNs that is followed by a second wave of monocyte recruitment. PMN-derived HBP is a potent chemoattractant for this wave of monocytes. HBP possesses alternating hydrophobic and hydrophilic domains within its structure, and hydrophobic stretches typically bind to lipid bilayers, thus explaining why HBP exhibits affinity for cell membranes and possesses chemotactic effects [13]. HBP directly activates the C-C motif chemokine receptor 2 (CCR2) present on monocytes [48], and this activation mediates the recruitment of monocytes and propagates inflammation and tissue damage [49] (Figure 3). Additionally, HBP is not a general amplifier of LPS-induced monocyte activation, and instead, it targets the production of a distinct set of mediators such as proinflammatory cytokines [50]. The binding of HBP to ECs not only causes increased endothelial permeability but also enhances ECs to secrete chemokines such as monocyte chemotactic protein 1 (MCP-1), the ligand of CCR2, to promote monocyte migration to the infectious loci [51]. Further experiments determined that HBP induces MCP-1 expression via a sequential activation of the FAK/PI3K/AKT pathway and the p38 MAPK/NF-*κ*B axis [51]. Other studies also suggest that HBP binds to a receptor expressed on monocytes, and the function of this receptor is dependent upon divalent cations and is possibly related to the scavenger receptor [52].

When monocytes become activated, these cells begin to move and roll on the ECs and to migrate to the infectious loci through the gaps between endothelial cells. HBP is capable of upregulating adhesion molecules such as ICAM-1, VCAM, and E-selectin on the endothelia, thereby promoting PMN-endothelial and monocyte-endothelial interactions [53]. HBP that accumulates and is deposited on the surface of ECs is also capable of triggering calcium-dependent activation responses in monocytes that ultimately result in increased firm arrest on the endothelia [54]. Interestingly, the identity and kinetics underlying the upregulation of these specific adhesion molecules are dependent upon the endothelial cell type, suggesting that adhesion molecules present on endothelial cells from different vascular beds are differentially regulated by HBP [53].

In other types of cells, a chemoattractant role for HBP was also observed. For example, in corneal wound healing, HBP interacts with G protein-coupled receptors (GPCR) to activate the PKC signaling cascade through the PKC*δ* isoform, subsequently leading to corneal epithelial cell migration [15]. Additionally, HBP has also been reported to support migration and proliferation of smooth muscle cells *in vitro*, and smooth muscle cells treated with HBP express higher levels of ICAM-1 [16].

## 3. HBP in Various Diseases

### 3.1. HBP in the Sepsis

Infectious diseases continue to pose a significant problem in terms of global healthcare, and sepsis is a common cause of morbidity and mortality in hospitals [55]. Kumar et al. [56] found that in patients suffering from septic shock, mortality was correlated with the time between the fall in systolic blood pressure and the initiation of effective antimicrobial treatment. Thus, it is imperative to detect severe infections and sepsis at an early stage to allow for improved treatment options. In sepsis, the molecular mechanisms underlying the induction of capillary endothelial leakage are of crucial importance. Severe sepsis is characterized by an uncontrolled increase in vascular permeability that results in hypotension, disturbed microcirculation, hypoxia, and organ dysfunction. As mentioned above, HBP is an inflammatory mediator that possesses the ability to induce vascular leakage [30] (Table 1). HBP proven to be a valuable diagnostic marker for suspected sepsis [57] has been demonstrated excellent prognostic and discriminatory properties in detecting the most severely ill patients suffering from sepsis [58] (Table 2). Experimental and clinical evidence supports a prominent role for this protein in the pathophysiology of sepsis-induced organ dysfunction [3].

#### 3.1.1. HBP and Circulatory Failure

A prospective study of 233 febrile adult patients with a suspected infection was conducted to investigate the usefulness of HBP plasma levels for predicting and diagnosing cases of severe sepsis with circulatory failure [59]. Patients were classified into 5 groups based on systemic inflammatory response syndrome criteria, organ failure, and final diagnosis. Of these patients, 26 exhibited a more serious circulatory failure that was refractory to fluid treatment and was thus defined as septic shock. Of the remaining patients, 44 had severe sepsis without septic shock, 100 patients had sepsis, 43 patients had an infection without sepsis, and 20 patients had an inflammatory response caused by a noninfectious disease. Plasma HBP levels of ≥15 ng/mL served as a better indicator of severe sepsis (with or without septic shock) than any other laboratory parameter investigated, and these other parameters included procalcitonin (PCT), IL-6, C-reactive protein (CRP), white blood cell count (WBC), and lactate (sensitivity, 87.1%; specificity, 95.1%; positive predictive value, 88.4%; negative predictive value, 94.5%). Thirty-two of the 70 patients diagnosed with severe sepsis were sampled for up to 12 h before signs of circulatory failure appeared, and in 29 of these patients, HBP plasma concentrations were already elevated. These results suggest that the prompt institution of adequate supportive treatment in febrile patients with increased plasma levels of HBP would likely reduce the risk of developing circulatory failure.

Subsequently, a number of studies verified that plasma HBP levels were significantly elevated in sepsis associated with circulatory failure [60, 61]. In a prospective study conducted using two patient cohorts treated in an intensive care unit [61], the data also demonstrated that HBP was associated with severity of disease, and an elevated HBP at admission was associated with an increased risk of death (an increased case-fatality rate at 28 days). HBP levels that rose over time may be indicative of patients with a deteriorating prognosis. Therefore, repeated HBP measurement in the ICU may help monitor treatment and predict outcome in patients with severe infections. A further study [62] in an international multicenter setting that included >800 patients at six different emergency departments in Sweden, USA, and Canada confirmed that compared to other biomarkers, the heparin-binding protein was the best predictor of progression to organ dysfunction (area under the receiver operating characteristic curve = 0.80).

To investigate the causality and mechanisms of action of HBP, a cohort study of patients diagnosed with septic shock from a randomized controlled multicenter study was performed [43]. Plasma HBP concentrations were weakly associated with fluid overload during the first 4 days of septic shock. In mice, intravenous injection of recombinant human HBP induced a lung injury similar to that observed after lipopolysaccharide injection. HBP increased the permeability of vascular endothelial cell monolayers *in vitro*. Additionally, interaction with luminal GAGs and activation of the PKC and Rho-kinase pathways mediated the permeability-increasing effect of HBP on endothelial cells.

#### 3.1.2. HBP and Acute Kidney Injury

Sepsis is the most common cause of acute kidney injury (AKI) [63]. Sepsis-induced AKI is caused by a combination of multiple mechanisms, including inadequate vascular leakage/perfusion, local tubular inflammation, and cell cycle arrest [55]. Of these factors, significant tissue inflammation within the kidney appears to be a critical mediator of sepsis-induced AKI [64]. HBP has recently been suggested to be involved in the pathophysiology of AKI according to data from a murine model and from human renal tubular epithelial cells [34]. Plasma HBP levels were significantly higher in 296 septic shock patients diagnosed with AKI and in those requiring RRT [34]. HBP levels identified patients suffering from moderate AKI with an area under the curve (AUC) of 0.85. Plasma concentrations of HBP from 245 patients taken upon admission to ICU (including 59 patients with severe sepsis) were also associated with the development of severe kidney injury [65]. Additionally, Tverring et al. [66] proposed that measuring HBP upon admission to the ICU added predictive value to known clinical risk factors associated with septic AKI.

In the septic animal models, HBP expression particularly increased at 24 h postinjury, and this expression decreased over the following hours. HBP played an essential role in the initial inflammatory reaction associated with sepsis-induced AKI, presumably by activating M1 macrophages and by suppressing TNF-*α* and IL-6 secretion [67]. Unfractionated and low molecular weight heparin blocked HBP-induced endothelial cell permeability [43] and renal tubular cell inflammation [34] in an *in vitro* murine model. Suppression of HBP expression by heparin injection following the development of AKI in septic mice resulted in a reduction in renal injury severity that was accompanied by a significantly decreased macrophage infiltration and activation [67]. Albumin, an established colloidal plasma expander in septic shock, however, could inhibit HBP-induced endothelial cell permeability while paradoxically increasing *in vitro* renal inflammation [68]. Therefore, strategies that limit early macrophage infiltration or activation may represent a novel approach in the prevention or treatment of AKI in septic patients.

#### 3.1.3. HBP and Acute Lung Injury/Acute Respiratory Distress Syndrome

Acute lung injury (ALI)/acute respiratory distress syndrome (ARDS) is a common cause of life-threatening acute respiratory failure in ICUs worldwide. ALI/ARDS is characterized by systemic inflammation, disruption of endothelial and alveolar epithelial barriers, and an increase in microvascular permeability, which together result in pulmonary edema and respiratory failure. Vascular hyperpermeability is one of the most essential pathophysiological processes underlying ARDS, and there are numerous clinical studies that focus on HBP and ARDS.

In a transfusion-related acute lung injury (TRALI) study [69], substantial amounts of HBP were released within 30 minutes of stimulation by human antibodies, although other soluble mediators, such as TNF-*α* and IL-6, were not released during the same time period. Additionally, the release of HBP was mediated via signaling pathways that involved Fc*γ*RIIIb and Fc*γ*RIIa. Based on this, HBP appeared to be one of the primary effector molecules of antibody-mediated nonhemolytic transfusion reactions, including those mediated by TRALI. Lin et al. [70] demonstrated that ALI/ARDS exhibited significantly higher median levels of HBP compared to those of cardiogenic pulmonary edema patients (17.15 (11.95 to 24.07) ng/mL vs. 9.50 (7.98 to 12.18) ng/mL, *P* < 0.001). Additionally, HBP levels in nonsurvivors were significantly higher than those of survivors (23.90 (14.81 to 32.45) ng/mL vs. 16.01 (10.97 to 21.06) ng/mL, *P* = 0.012). Multivariate logistic regression analysis revealed that HBP at enrollment was the independent predictor for 30-day mortality (odds ratio = 1.52, *P* = 0.034), indicating that HBP may prove to be a good drug target for therapeutic intervention. Johansson et al. [71] revealed that a correlation exists between HBP levels and the development of ARDS after trauma (*P* = 0.026, *n* = 47), indicating that HBP may serve as a biomarker candidate for early detection of ARDS development after trauma. Similarly, Kaukonen et al. [14] found that the concentration of HBP was markedly elevated in all critically ill patients diagnosed with influenza A (H1N1) infection, even in the presence of a low white cell count. HBP concentrations correlated with the lowest ratio of the partial pressure of oxygen in arterial blood to a fraction of inspired oxygen (PF ratio) during the ICU stay, indicating HBP levels were associated with more pronounced respiratory dysfunction. In another recent study, Tyden et al. [72] also observed an association between elevated HBP levels in plasma and circulatory and respiratory failure.

Additionally, Liu and colleagues [73] used an animal model to examine the role of HBP in ARDS. In ARDS animals, HBP levels dramatically increased and showed significant correlation with lung wet/dry ratio and BALF total proteins. As lung wet/dry ratio and BALF total proteins reflected impairment of the alveolar-capillary barrier, these results also suggested that HBP was positively correlated with lung injury severity. Both the inhibition of HBP function and HBP endotracheal instillation demonstrated that HBP played an important role in the alteration of lung vascular permeability in ARDS. Additionally, HBP released from PMNs was a *β*2 integrin-PI3K signaling pathway-dependent process that presented a potential novel therapeutic target for ARDS treatment.

### 3.2. HBP in Bacterial Skin Infection

Necrotizing fasciitis is a surgical diagnosis characterized by friability of the superficial fascia, dishwater-gray exudate, and a notable absence of pus [74]. Mortality is higher among patients who develop streptococcal toxic shock syndrome or septic shock (38% and 45%, respectively). Herwald et al. [11] reported that M protein released from the streptococcal surface could form complexes with fibrinogen, and these complexes triggered the secretion of HBP from human PMNs. M protein also acted as a potent activator of monocytes, causing these cells to express the cytokines IL-6, IL-1*β*, and TNF-*α*. This response was significantly enhanced in the presence of HBP. Further analyses of tissue biopsies taken from patients suffering from necrotizing fasciitis or severe cellulitis caused by *S. pyogenes* bacteria (M1 serotype) revealed that the recruitment of PMNs and monocytes/macrophages to the infectious focus was associated with the release of HBP [75]. The results showed that M protein, in synergy with HBP, evoked an inflammatory response that may contribute to the profound pathophysiological consequences observed in severe streptococcal infections. Additionally, cultures together with serological data indicated that the group A streptococcus was the predominant etiological agent responsible for spreading superficial skin infections such as erysipelas. A more detailed study [76] involving 12 patients diagnosed with erysipelas showed increased levels of HBP in the infected areas compared to levels observed in the noninfected areas. Meanwhile, group A streptococci induced HBP release during skin infection, and this may lead to erysipelas symptoms. These studies suggested that HBP could play an essential role in the edema formation observed in streptococcal skin infections.

Thus, Lundqvist et al. [77] found significantly increased HBP levels in wound fluid derived from chronic leg ulcers, and they demonstrated that secreted products from Pseudomonas aeruginosa could induce the release of HBP from human neutrophils. The data suggested a possible link between bacterial presence and HBP release in chronic ulcers. Additionally, high HBP levels that resulted in endothelial hyperpermeability and neutrophil recruitment may represent an early pathogenic step during the development of ulcers. Therefore, these results indicated a novel mechanism by which *P. aeruginosa* may aggravate inflammation in chronic leg ulcers and could be helpful in the development of novel therapeutic strategies.

### 3.3. HBP in Acute Bacterial Meningitis

Acute bacterial meningitis (ABM) is a medical emergency and may cause substantial neurologic sequelae [78]. *Streptococcus pneumoniae* and *Neisseria meningitides* are the primary pathogens in adults, and these infections result in an overall mortality rate of approximately 30% and 10%, respectively. Early treatment with antibiotics and dexamethasone improves prognosis, and therefore, rapid diagnosis is critical.

Two patient cohort studies (one prospective and one retrospective patient cohort) were performed that incorporated 174 patients suffering from suspected central nervous system infections [79]. Heparin-binding protein levels were significantly higher (*P* < 0.01) in patients with ABM (median 376 ng/mL, range 12–858 ng/mL) than those observed in patients diagnosed with viral central nervous system infection (median 4.7 ng/mL, range 3.0–41 ng/mL), neuroborreliosis (median 3.6 ng/mL, range 3.2–10 ng/mL), or in control patients with a normal cerebrospinal fluid cell count (median 3.5 ng/mL, range 2.4–8.7 ng/mL). In the prospectively studied group, an HBP concentration exceeding 20 ng/mL resulted in a sensitivity of 100%, a specificity of 99.2%, and positive and negative predictive values of 96.2% and 100%, respectively, for the diagnosis of acute bacterial meningitis. The area under the receiver-operating characteristic curve for heparin-binding protein was 0.994, which was higher than those of the other investigated parameters. Linder et al. [79] concluded that measuring HBP in CSF from patients with suspected meningitis could improve the diagnostic accuracy for differentiating between bacterial and viral central nervous system infection, ultimately allowing the clinician to begin adequate treatment earlier. This was the first study investigating the presence of neutrophil-derived HBP in the CSF.

A recent study [80] in 2018 concurred with and confirmed these results. This study was the first study to investigate serum HBP in meningitis and to correlate between CSF and serum HBP. The mean serum level was 192.2 ± 56.6 ng/mL in bacterial meningitis, 3.7 ± 1.9 ng/mL in viral meningitis, and 0.84 ± 0.3 ng/mL in the controls. HBP levels in both the cerebrospinal fluid and the serum were significantly higher in patients with bacterial meningitis. Cutoffs of 56.7 ng/mL and 45.3 ng/mL in cerebrospinal fluid and serum, respectively, showed 100% overall accuracy. Even in patients who received prior antibiotics, these levels remained elevated. Further research to allow for serial measurements of HBP plasma levels for close monitoring of critically ill meningitis patients, instead of the repeated lumbar punctures that are currently used in the follow-up of acute bacterial meningitis cases, is recommended.

### 3.4. HBP in Other Infectious Diseases

In addition to bacterial infection, Vieira et al. [32] reported that leptospiral agents could induce HBP release through a controlled degranulation mechanism that was dependent on various cell signaling pathways and Ca^2+^ influx. Unlike streptococcal M protein, however, Leptospira-induced HBP release was not mediated by fibrinogen and *β*2 integrins. These findings possessed clinical implications, as high levels of HBP were detected in serum obtained from patients with leptospirosis, particularly at the early phase of the disease. In general, these findings described a new mechanism by which leptospirosis-induced pathophysiological complications may arise, and they proposed HBP as a novel early diagnostic marker for human leptospirosis.

HBP was also involved in infectious diseases caused by protozoan parasites that cause important diseases in humans. HBP present on the surface of infectious forms of *Trypanosoma cruzi* was involved in the adhesion of amastigotes and epimastigotes to the host cells and the intestinal epithelium of triatomines, respectively [81–83]. Certain studies provided evidence of the expression of HBP in *Leishmania braziliensis* promastigotes, thus suggesting the participation of HBP in the interaction of the parasite with intestinal cells in *Lutzomyia* species and in the life cycle of the parasite [84–86]. Similarly, Martins et al. [87] verified the presence of HBP in *L. chagasi* promastigotes, and they demonstrated that HBP spread over the outer parasite surface and internally adjacent to the kinetoplast. As observed during the *T. cruzi* infection process [83], HBPLc may act as an additional virulence factor that contributed to the recognition and entry of the parasite into macrophages and that triggered the process of infection, as blocking of this function with heparin generated a partial reduction in the internalization of *Leishmania* by RAW macrophages *in vitro*.

### 3.5. HBP in Noninfectious Disease

The current findings supported the idea that inflammation played a part in the pathophysiological mechanisms involved in ST-segment elevation myocardial infarction (STEMI) [88]. Two recent animal studies demonstrated the role of inflammation and neutrophils in the atherosclerotic process, in healing after myocardial infarction, and in cardiac remodeling [89, 90]. Ipek et al. [91] proposed that HBP levels were significantly higher in patients diagnosed with STEMI compared to those of healthy controls (18.07 ± 13.99 versus 10.09 ± 5.29 ng/mL, respectively). In a receiver-operating characteristic curve analysis, an HBP cutoff level of >11.46 ng/mL exhibited 74% sensitivity and 58% specificity in predicting myocardial infarction. HBP levels were positively correlated with the thrombolysis in myocardial infarction (TIMI) score (*r* = 0.651). In multivariate linear regression analysis, the TIMI score was an independent predictor of the HBP level.

In summary, HBP was an infection marker that may be important in patients with STEMI. In addition to STEMI, HBP levels exhibited a slower increase in a rabbit model of acute mesenteric ischemia (AMI) compared to that of with CRP and IL-6 [92]; however, diagnostic sensitivity and specificity should be evaluated in further clinical trials.

## 4. Conclusion

Heparin-binding protein is released immediately upon neutrophil stimulation by multiple mechanisms. This protein is an inflammatory mediator and potent inducer of vascular leakage. Therefore, the level of HBP is associated with the development of infectious diseases, particularly in bacterial infections that result in sepsis. As a valuable diagnostic marker, HBP has proven to possess excellent prognostic and discriminatory properties in regard to detecting bacterial infection with high sensitivity and specificity. HBP may also provide a new target for the treatment of bacterial infection. There are, however, still many gaps in the research regarding the molecular mechanisms and the clinical applications of HBP *in vivo*, and further exploration and efforts by researchers are required to clarify these functions.

## Figures and Tables

**Table 1:** The molecular mechanisms of HBP in various diseases.

Disease	Molecular mechanisms	Reference
Sepsis		
Circulatory failure	HBP interacts with GAGs and activates the PKC and Rho-kinase pathways to increase endothelial cell permeability.	[43]
AKI	HBP activates M1 macrophages during the initial inflammation response, and suppression of HBP expression by heparin injection in septic mice results in a reduction in renal injury severity.	[67]
ALI/ARDS	HBP levels dramatically increase and exhibit significant correlation with lung wet/dry ratio and BALF total proteins. Additionally, HBP plays an important role in the alteration of lung vascular permeability in ARDS.	[73]
Bacterial skin infection	M protein induces HBP release during skin infection, and this directly and indirectly contributes to a number of profound pathophysiological effects such as endothelial hyperpermeability and neutrophil recruitment.	[11, 75]
Leptospirosis	HBP is induced by leptospires and their secreted products through a controlled degranulation mechanism that is not mediated by fibrinogen and *β*2 integrins.	[32]
Protozoan parasites	HBP plays a vital role in the attachment and invasion process of a variety of intracellular pathogens, and HBP even participates in the life cycle of certain parasites.	[81–86]

**Table 2:** Clinical applications of HBP for various infectious diseases.

Disease	Function	Reference
Sepsis		
Circulatory failure	HBP is associated with severity of circulatory failure, and elevated HBP at admission is associated with an increased risk of death.	[59–62]
AKI	Plasma concentration of HBP is related to the development of severe kidney injury.	[34, 65–67]
ALI/ARDS	HBP is associated with ALI/ARDS.	[65, 72, 73]
Bacterial skin infection	Increased levels of HBP in the infected skin were observed when compared to those of the noninfected areas in patients suffering from bacterial skin infections.	[76]
Acute bacterial meningitis	HBP levels in cerebrospinal fluid and in serum were significantly higher in patients diagnosed with bacterial meningitis.	[79, 80]
Leptospirosis	High levels of HBP were detected in serum from patients diagnosed with leptospirosis, particularly at the early phase of the disease.	[32]

AKI: acute kidney injury; ALI: acute lung injury; ARDS: acute respiratory distress syndrome; HBP: heparin-binding protein.
